# Self-perception in Iranian adolescents with diabetes: a qualitative study

**DOI:** 10.1186/s40200-015-0163-0

**Published:** 2015-04-24

**Authors:** Shahnaz Rostami, Zahra Parsa-Yekta, Tahereh Najafi-Ghezeljeh, Zohreh Vanaki, Kourosh Zarea

**Affiliations:** Chronic Disease Care Research Centre, School of Nursing and Midwifery, Ahvaz Jundishapur University of Medical Sciences, Ahvaz, Iran; Nursing Care Research center, School of Nursing and Midwifery, Tehran University of Medical Sciences, Tehran, Iran; Department of Nursing, Faculty of Medical Sciences, Tarbiat Modares University, Tehran, Iran

**Keywords:** Adolescents with diabetes, Getting insight, Positive self-perception, Perception of being healthy, Life satisfaction

## Abstract

**Background:**

It is obvious that self-perception can play an important role in the development of self-management behaviours among adolescents with diabetes to promote their health and quality of life. This study seeks to explain self-perception in adolescents with diabetes.

**Method:**

This qualitative study, which is of “grounded theory” type, was performed in 2013 in Ahvaz, Iran, through semi-structured interviews with ten adolescents with type 1 diabetes, two parents and a nurse, who were chosen objectively. Data analysis was performed using Strauss and Corbin 1998 method.

**Results:**

Four main theme was obtained from the analysis of data, and the consequence theme was inferred as follows: getting insight (knowledge acquisition and belief management), perceiving similarities with others (not hiding the disease, showing the illness is normal, and accepting an active role in the family), and self-care management (independent control of food and treatment regimen and understanding of capabilities to manage the future of life and manage the daily activities of life), and life satisfaction (perception of being healthy and having a normal life).

**Conclusion:**

Getting insight into the disease is the most important part of perceiving similarities with others and offering self-care, which can provide a person’s positive perception of himself/herself and the illness, as well as life satisfaction for their adolescent over time. These results are an operational guide for personnel providing health care services, especially diabetes specialist nurses.

## Introduction

The prevalence of diabetes as a common metabolic disease is estimated at 2.8% in 2000 and 4.4% in 2030 for all age groups around the world [1,2]. As many psychological and cognitive changes may occur during puberty, the incidence of diabetes in this period makes challenging its management [3]. Patients who have difficulty in the control of diabetes often have negative self-concepts, feel hopeless and, therefore, become lax about following their regimen [4]. Effective diabetes self-management requires persons to be active participants in their care to prevent poor function and maintain independence persons’ perceptions and understanding of the potential impact of the disease could influence self-management practices and success [5]. Obviously, self-perception can play an important role in self-management behaviours among adolescents with diabetes. Perception of health can also affect self-care, diabetes management and some aspects of the quality of life in these adolescents. Perception of health is an aspect of health, which depends on whether there is a disease and on its impact on health-promoting behaviours, and is considered important by people [4]. Process of self-management begins with an internal negotiation between individual beliefs about health and disease, that is, the thing that faces a person with the threat of the disease and the need for self-management [6].

The management of diabetes is a great challenge especially in adolescents [7]. Self-perception changes dramatically during adolescence. Most self-descriptions of children focus on their objective physical characteristics, general activities and capabilities, while adolescents pay relatively more attention to social and personality characteristics and finally the belief and value systems as well as thought processes. Sometimes it is due to cognitive changes (such as change toward more abstract concepts) that these thoughts allow adolescents to infer the characteristics that cannot be easily observed [8]. Self-perception is an abstracted concept which that in a study by Whieler (2009), self-perception has emerged as one aspect of self-management theme [9]. Amer (2008) argues that the recognition of children’s perceptions of themselves is important to manage and understand their health in the long term, while many adults perceive diabetes mellitus in children as an overwhelming experience [10]. In his research, children usually had a positive self-perception and a good attitude towards the disease. Song (2009) states that people with a positive perception of their health status less take care of themselves because they feel less need to improve their health; and conversely, those with a negative perception of their health more take care of themselves because they think that self-care can reduce the risk of complications [4]. In addition, Huang et al. [11] argue that people with less worry pay less time to take care of their own health. Self-perception and reporting of health vary among different individuals with diabetes [11]. An understanding of adolescents about self is gradually achieved through a qualitative research. Perception of adolescents with type 1 diabetes has received little attention in research [3]. On the other hand, studies have shown that nurses have the key role in providing care for the patients in order to manage the metabolic condition of the patients [12].

It is certain that people’s perceptions are formed under the influence of social and cultural context as well as social structure, which in turn is reflected in the structure of families and health care systems. The vast and constant communications of adolescents and their families with health care providers and other social systems, including schools, can be effective in shaping their perceptions of illness and health. Therefore, a study of the perceptions of adolescents with diabetes in this particular socio-cultural context can help health care providers to improve health and ultimately their quality of life. However, differences in the self-perception of people with diabetes (in terms of age and disease) in various socio-cultural status, the lack of studies in this regard, and different results of various studies on the patients’ perception of health led us to perform a study aimed to explore the self-perception in adolescents with diabetes type 1.

## Methods

### Design

A qualitative research design with tenets from grounded theory was used to elicit detailed descriptions of adolescents’ perception of self. Semi-structured interviews were used to collect data from March 2010 to March 2011. Data were submitted to grounded theory as a qualitative approach, whereby the researchers sorted through large amounts of data, make inferences from the data on the basis of the context, provide new insights, and conceptualize realities presented in the text. Qualitative methods are considered more appropriate in areas of insufficient knowledge, yielding deeper insight than quantitative approaches [13].

### Participants

Participants were recruited using purposeful sampling from Tehran and Ahvaz city. The inclusion criteria for adolescent participants were that they be adolescents, as defined by the World Health Organization [14], have T1DM for at least two years, have no other chronic diseases, and be willing to share their experiences and perspectives. A theoretical purposive sampling technique was used to select participants. This sampling technique is concerned with choosing participants based on the emerging theoretical themes and concepts and not with the representativeness of the sample [13]. Ten adolescents (seven males and three females) with T1DM were recruited at two diabetes management clinics affiliated with two teaching hospitals in two urban areas of Iran. To learn about the perspectives of parents, two family members (two mothers) and one nurse that had provided care to adolescents with T1DM for a minimum of two years. Polit and beck (2014) believe that maximum variation sampling involves deliberately selecting cases with wide variation on dimensions of interest [15]. In this study sample included 10 patients aged between 12 and 19 year who were purposively selected to provide variation in gender, age, age of diagnosis and place of residency.

### Data collection

Individual semi-structured interviews (20–100 min) were held at the clinics or in participants’ homes. Key questions for the adolescents were “Will you please explain what you explain yourself and yours health?” and Follow-up questions, such as “are you know yourself healthy or patients?” were used as needed.

The key questions were for parents and nurse what you explain diabetes Interviews were conducted by the first author in Persian and tape-recorded. Interviews were conducted in diabetic centre. All participants were interviewed once; then, five adolescents consented to follow-up interviews. Keeping with the selected methodology, data collection and analysis proceeded concurrently, with interviews continuing until data saturation was achieved [16]. In this study saturation achieved after repeating the previous codes and not appearing any new codes also after theoretical saturation completed.

### Ethical considerations

Participants were informed of the study aims and methods, and were assured that participation was anonymous and voluntary, and that they could withdraw at any time without penalty. Written informed consent was obtained from all participants. The Tehran University of Medical Sciences ethics committee and research council approved the study proposal. Project number; 90/130/44, approval date; 2011/4/11

### Data analysis

Strauss’ and Corbin [13] constant comparative data method was used to analyse all data from the interviews and field notes. This method involves moving back and forth among data sets to discover themes or patterns and to determine variation of patterns. Interviews were transcribed verbatim and read through several times to produce an overall, general impression of the data. Data were simultaneously coded and analysed as data were collected. When possible, the adolescents’ own words were used in developing the categories. Coding involved reading every piece of information and giving meaning to all units of information. Units of information that related to each other were then categorized. Categories, subcategories, and themes expressing the latent content of the text were developed through code comparison. As categories surfaced, the researcher then compared categories with other categories and units of information for each participant; participants were also compared with other participants. The analysis process conducted based three Strauss and Corbin stages included; Open coding, axial coding and selective coding [17]. Four researcher were involved in data analysis and all of them expertise in qualitative research and qualified in nursing. At the first stage of analysis 440 codes derived from interviews, then 37 categories revealed and after that merging similar categories, 4 main theme emerged finally. Categories were compiled using the MAXQDA-10 software (VERBI GmbH, Berlin, Germany) to provide later retrieval and to document the cycles of the reflection of each interpreter throughout the analysis.

### Rigor

Trustworthiness is established when the reader can audit researchers [18]. This position was proposed by Lincoln and Guba (1985), who created standards for the trustworthiness of qualitative research that parallel the standards of reliability and validity in quantitative research [15]. On this way, peer checking was conducted to confirm the credibility of data. The data were coded and categorized independently by the researchers; in the case of disagreement over categories and themes, the researchers discussed the content until consensus was reached. A summary of the transcripts and the related analysis was given to five participants, who confirmed the accuracy of the analysis. Dependability of data analysis was achieved by an audit trail, which was maintained throughout the data collection [19]. Researchers [18].

## Results

Results showed that the mean age of the adolescents was 15 years, and an average of 5 years has elapsed since their diagnosis. In other words, the participants’ disease was diagnosed at the age of 10 years. One parent had a high school diploma, and another held a bachelor’s degree. Nurses held a B.Sc. degree (Table 1 and Table 2). Data analysis revealed three major themes: getting insight into the disease, perceiving similarities with others and managing self-care. These themes indicate that after getting insight into the disease, diabetic adolescents got to perceived similarities with other adolescents, which represent a positive attitude to the disease in a way that it improves their self-management and life satisfaction (perception of being healthy and having a normal life) (Table 3).

**Table 1:** **Demographic characteristic of adolescents with diabetes**

**Participant No**	**Ethnicity**	**Educational background**	**Sex**	**Mean age at the time of interview**	**Mean age at the time of diagnosis**	**Birth order**
1	Fars	High school diploma	Male	18	16	8
2	Arab	Junior high school	Male	16	2	2
3	Fars	High school diploma	Female	17	10	1
4	Arab	High school diploma	Male	17	13	5
5	Fars	Student	Male	19	14	3
6	Arab	Middle school	Male	12	10	2
7	Arab	Middle school	Male	15	9	2
8	Fars	Middle school	Female	13	11	4
9	Arab	Middle school	Male	13	7	3
10	Fars	Middle school	Female	15	10	1

**Table 2:** **Demographic characteristic of other participants**

**Participant No**	**Relationship with adolescents**	**Educational background**
11	Mother	High school diploma
12	Mother	Bachelor’s degree
13	Nurse	Associate degree

**Table 3:** **Main theme and sub-theme**

**Main theme**	**Sub-theme**
Getting insight into the disease	Acquisition of knowledge
	Management of their beliefs
Perceiving similarities with others	Not hiding the disease
	Showing that the disease is normal
	Accepting an active role in the family
Self-care Management	Independent self-control
	Management of the daily life activities
	Understanding of capabilities to manage the future of life.
Life Satisfaction	Absence of serious complications of the disease and treatment
	Doing daily activities
	Normal levels of blood sugar

### Getting insight into the disease

This theme has derived from the following two sub-theme: acquisition of knowledge and management of their beliefs. Getting insight is the start of an event and an important basic psychological-social process for the perception of the disease.

*****Since the time of diagnosis, nurses and physicians are responsible for informing them and begin the training.

After this, the patients themselves and their families have looked for practical information on self-care and, ultimately, have reduced problems. The nurse, who participated in the study, said: “*For parents, however, we try as far as possible to make it easier for them to accept this. We explain a little to them that it is so easy to deal with this disease if they raise their awareness, accept and learn all that they are told, and then they can easily control this disease. We recommend them to accept the treatment*”(13)

A 17-year-old girl also stated: “*At that time (the first day of diagnosis), it was hard a little for me because my knowledge was moderate. It was a little hard for me to accept that I am diabetic. But after I was hospitalized, I was explained about the disease and what should I do. So, I compromised and accepted it*”(3).

The families’ passion to get information has helped the patients to accept the disease in the way that in a note in the field, the researcher observed that “*… before and after the interview, even when the researcher was being fare welled, the mother was seeking answers to questions that makes the life of her child with diabetes easy, and she was adamantly speaking to get information and sought the support of the sources of social support such as help from health centres, schools and associations, and she was asking how to regularly receive magazine of the Diabetes Association and attend the conferences* ”(8).

Finally, she has been useful and important to get information from multiple sources of information for adolescents, and has been assisting them to get insight and ultimately accept the disease.

Another important issue that had helped getting insight into the nature of the illness was the fact that they were enjoying their religious beliefs and were trying to gain comfort and hope through reading the Quran and prayer, participating in religious ceremonies, and resorting to the Holy Imams (Q), and thus it has been a factor to facilitate the acceptance of the disease. Thus, another subclass is inferred as the management of their and their families’ beliefs.

A participant stated, “*I don’t caused the illness myself; it is the divine will*” (10). This religious view has caused the patient to submit to the will of God.

Thus, the hope of cure has facilitated the acceptance and endurance of the disease.

A 15-year-old boy stated that “*In addition to prayer, the very insulin has kept us alive*” (8).

Over time, adolescents with religious beliefs had less fear and anxiety, and most importantly, they gained insight (deep knowledge) into the disease, after getting information from the first staff of the health team and using their advice so that they knew how to deal with it, which facilitated the acceptance of their illness and how to care for them.

A 19-year-old teenager said: “*It means I know something I need to know about diet, insulin and exercise and its effect*”(5).

Another said: “*I learned about the disease. I learned to always use insulin because my body does not produce insulin*”(8).

### Perceiving similarities with others

This class has derived from the following three sub-classes: not hiding the disease, showing that the disease is normal, and accepting an active role in the family.

***** Following the acquisition of knowledge about the disease and how to deal with it, fewer adolescents tend to hide the illness from others. They found that low blood sugar is a perennial problem that must be controlled by them, and that helping others, especially in school is very important. Therefore, they have less hidden it.

A 17-year-old girl stated: “*Well, I might get in trouble, but I can use the other to help me by making informed them, for example, friends, teachers …*” (3) and another person said, “*everyone knows it; friends treat me like the rest*”(2).

And, the others’ awareness of the disease and their specific conditions are not important for them.

***** After Getting insight into the disease, one of the most important efforts have been to show their disease is normal and to avoid exaggerating it for themselves, which have been also supported by their families, so that, one of the participating mothers said, “*I treat him like my other children*” (7).

Another adolescent also said, “*for example, I treated myself so that I am like other people. For example, I have no horns, no tail; I’m like the others, my blood sugar is just a little high*”(8).

Another participant provides a better understanding of his disease compared with other diseases, and says: “… *there are other diseases that are much worse. I kind of think diabetes is better than them*”(4).

Thus, it can be seen that, since the adolescents have considered their disease to be normal, this perception is an important factor to maintain similarities with others and tend to have a normal life like other adolescents.

Participants described diabetes as part of themselves and their own lives. *“My illness is part of my existence. It has its own goodness and badness”,* states a 13-year-old girl (8)*.*

***** In this regard, the active role that the adolescents accept in the family in this class indicates their opinion that their disease is not a limiting factor for themselves and tried to continue the normal routine of their lives. It is obvious that when the adolescents undertake some roles in the family to treat their disease as a normal state, these roles may vary depending on their age and sex. A 15-year-old girl says, *“I sweep the house, I wash the dishes, I take care of my sister, I do my dad’s work”* (10). Whereas a 17-year-old boy says, *“at home … I go downstairs and open the dairy shop”* (4).

### Self-care management

This class has derived from the following three subclasses: independent self-control, management of the daily life activities, and understanding of capabilities to manage the future of life.

***** After getting insight into the disease and knowledge about how to care for them, the adolescents achieved independent self-control over time due to continuous compliance with the principles of care and have been able to rely on themselves in offering care. A 19-year-old boy says about it, *“I went to the general doctor alone, saying that sir, write me a haemoglobin HGA1C. I went to the lab, I saw the results myself. If it was high, I was trying, for example, to decrease it. I did not go to the doctor. When I saw the result, I myself could understand it. The last time was in May when I was tested, it was 6.8, while it is said to be below 7 for a diabetic, I got the test three days ago. It was 5.8”* (5).

Another participant is a 17 year-old teen girl, *“… if I do an activity like exercise, I take less insulin depending on the exercise I do. Then, I test my blood sugar every time to prevent suffering from a drop in blood sugar because of the exercise. Well, my diet is a little different. Because some days, when I’m at home not at school, I do less activity. That is why my insulin sometimes may be a little bit more.”* (3).

The adolescents’ self-reliance to regulate blood sugar over time leads to take “care management” in a way that they are able to decide in different situations.

*“I know in my mind how much should I take. I know its amounts. Maybe somehow I can better decide on a diet than health care personnel. My meal plan must be constantly monitored, and I constantly think about the food that I eat. Whenever I gave my three-month blood sugar test, it was normal, that is, my blood sugar returned to its normal level from that time. It was like an ordinary person till now that two years go. I never felt that I cannot regulate my blood sugar, and I can encounter many problems. I was much more comfortable with it”*, states a participant (5)*.*

**** Given that all of them are engaged in education, all daily life activities of the adolescents were assigned to these people, who alone have been responsible for doing it.

*“From when I was a kid, there was nobody who wants, to say, to help me in lessons, I myself did my homework”,* says a 13-year-old girl (8).

It is important to say that, over time, the adolescents with diabetes have learned to do well their daily life activities by offering self-care and have tried to take over the management of their lives. The same student continues, *“I have learned to always use insulin, I must have a lot of activities. Usually, I play volleyball, badminton, swimming. When I come back home, I measure my blood sugar. I go to weddings and birthday parties, to be happy and not think about the disease. Each morning when I wake up … when I wash my face and hands, I give my test before breakfast. Then, for example, if it was low, I don’t use insulin, but if it is high, I take insulin, and then eat my breakfast.” (12)*

Another adolescent sates, *“Now that I have blood sugar, it was good for me because exercise entered my life. I had no particular difference with the time when I did not have diabetes; I’m just a little more regular. I already did not eat the breakfast too much, but now I have to eat it”* (5). Another participant also confirms this, *“… I was able to do daily activities. If I continue my studies it can convince me. I was able to do other activities. I have a somewhat normal life and have no problem with daily activities.”* (1). Participants managed their care with daily activities and preventive activities. A 17-year-old girl states, *“My life is like the lives of all ordinary people. The only difference is that when I wake up every morning, I have insulin injection and then must do my meal plan and activities according to a special diet”* (3).

Management of all aspects of self-care has resulted in having no difficulty from the disease, treatment and life. The same 17-year-old girl says, *“It is much easier than other diseases … well, it is correct that it has some complications, but if you take care of it, you can avoid the complications too.”* (3).

Another participant says, *“During the past few years, I know how to do my business … the life is related to me, I know how to do my business. I do a three-month blood sugar test myself … and just there is no other thing that I want to learn. It means I know something I need to know about diet, insulin and exercise I learned from time”* (5).

Thus, it can be seen that, over time, the adolescents have tried to manage the activities related to education and to establish specific conditions of living with the disease and regulate their lives.

***** In addition, it is important that they have gained this perception that they can also plan for the future of their lives and believe that the disease does not affect future decisions. Therefore, another subclass was obtained: understanding of capabilities to manage the future of life.

*“Diabetes does not affect the decisions that I take. I can somehow decide and do my own personal work. I liked computer science. For a long time, I liked it and now I’m interested in it too. I kind of say that the computer course is the only thing that I want to study at university fondly”,* states an 18-year-old boy (1)*.*

As it was stated, the adolescents believed that the disease does not affect their educational and professional future. One participant clearly states, *“I don’t think this disease can affect the decision to choose my university course, or whether or not I go on the course”* (10).

It is well seen that after the acquisition of positive perception of themselves and the illness, the adolescents have found the capability necessary to manage their disease over time in a way that they have no serious problems in life due to proper performance in self-care. In other words, they have a normal life and are satisfied with it. This was inferred as a consequence of this study. Here are some comments from the participants who have referred more explicitly to the consequence.

### Life satisfaction

Sense of life satisfaction in adolescents, which is followed by the absence of serious complications of the disease and treatment, feeling of being healthy, *normal levels of blood sugar, and doing daily activities are important indicators that, if achieved, can show life satisfac*tion.

A 15-year-old girl expresses his feelings of the life, *‘in general, I have a happy life. It is a bit turbulent, but I’m fine“* (10). Another person also states the feeling of being healthy, *“I have just the same healthy body. Thank God, I consider myself healthy.”* (1). Due to the exact compliance with care measures, adolescents with diabetes in this study were not physically challenged and therefore had experienced the “health”. A female participant states, *“I do not think the life of a diabetic is much different from the life of a healthy person. I think they only might be a bit healthier than non-diabetics because they know they are sick; and because of this, they feed better than others.”* (3). That is why they were satisfied with themselves for self-care activities because their blood sugar had been well regulated. An 18-year-old boy states, *“Thank God that my blood sugar level has been well regulated. I’m very happy with myself, I am happier with my doctor … and I am satisfied with my life too.”* (1).

Even repeated injections have not caused discontent because they have brought their health status. One participant expressed, *“I must take insulin for the rest of your life, and am also not afraid of it too.”* (10).

It is obvious that, since controlling blood sugar can prevent it from causing major physical problems for adolescents, it, in turn, not only can generate perceptions of feeling of “being healthy” for them, but also they can easily continue the daily activities of their life. One participant says, *“… the disease did not interfere with my life. I see no difference there. It is not cumbersome for me.”* (6). A 17-year-old girl stated, *“My life, like the lives of ordinary people … I do not think the life of a diabetic is more different than a healthy person”* (3). Another girl stated her experience with the disease, *“No changes have been created in our lifestyle and our relationship due to disease … my blood sugar is just a little high. For this reason, I observe the prescriptions so that I become like normal persons”* (7, 8).

Thus, as was stated at the beginning, the adolescents have been able to perform their daily activities because they have no particular problem in their physical condition, and more importantly, the continuous infusion of the drug has been normal for them and … all these circumstances led them to achieve life satisfaction.

Although the sensitivity created after the disease may be regarded as natural, it becomes a proper treatment when people have a positive perception and feeling about themselves and their disease.

A 17-year-old girl stated that, *“… but well … if you look at it from a different angle, in my opinion, it cannot be considered as a disease. It is much easier than other diseases … well, it is correct that it has some complications, but if you take care of it, you can avoid the complications too.”* (3).

Another participant is a 19-year-old boy who says, *"… (the disease) did not interfere with my life. Furthermore, I see no difference there. It is not cumbersome for me (5).”*

**Figure 1:**
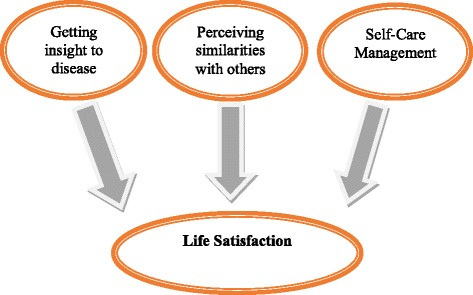
Self-perception in diabetic’s Iranian adolescents. This figure show that how life satisfaction in Iranian adolescences with diabetes formed. Getting insight into the disease, perceiving similarity and self-care management coming together then result to life satisfaction.

This positive perception of the disease and how to deal with it has led the adolescents to not regard themselves as patient, and regard themselves as non-diabetic teens. A 13-year-old girl says, *“That’s not to say that since I am a diabetic, I’m different from them (friends /classmates) … my appearance is not different from theirs because we all are the same and are not different from each other in spiritual, moral, scientific and from all aspects“* (8). Another says, *“I do not think the life of a diabetic is more different than a healthy person”* (3). The relationship between the above themes is shown in Figure 1.

## Discussion

This study was conducted for answering the question, what is the self-perception of adolescents with diabetes? The results showed that after getting insight and knowledge about the disease, adolescents with diabetes considered it to be a part of them and showed that the disease is normal. In addition, as a result of self-care management, they believed to be similar with healthy people. Therefore, they have gained this perception that, despite the disease, they can manage their daily lives over time. Also, they have gained the self-efficacy by which they can also decide the future of their lives. Responsibility in personal work and independent self-care show that adolescents have gained a positive perception of the illness and considered themselves healthy. In this case, they also have a life satisfaction.

Unlike our study, some of the participants in the study by Olshansky described themselves as diabetics who had a good understanding of living with the disease [20]. In line with our study, Kadohiro, however, stated that adolescents regarded diabetes as part of their lives [21]. In addition, Moreira and Dupas stated that children identified diabetes as part of themselves, and therefore accepted it [22].

In this study, adolescents with diabetes regarded themselves similar with their peers. Feeling of being different from others is worrisome for adolescents with type 1 diabetes [23-25]. Huus and Enskar also reported that adolescents with type 1 diabetes described their life experiences as a pendulum swinging between normal and abnormal conditions [26]. But in the study of Miatton et al., children with surgically repaired congenital heart disease did not report differences in the perceived competence or worry relative to healthy peers [27].

In agreement with our study, children generally had a positive self-perception and a good and very positive attitude towards the disease in the study by Amer [10]. Also, Sparud-Lundin et al. stated that adolescents have been trying to determine their identity as a healthy person [28]. In a study by Leung et al., perception of the disease in people with diabetes was reported to be extremely negative and was associated with lower self-concept and depression [29]. The perception of self-health and mental welfare were reported to be poor in the study by Huang et al. [11]. Esteban y Peña reported that the perceived health in individuals with diabetes (64.12%) is weaker than non-diabetics (38.57%.) [30].

In this study, adolescents felt healthy and considered themselves healthy. Of course, as an invisible and abstract concept, health is the result of assumptions by people, which goes back to their physical, psychological and social situation. According to new definitions, it refers to the enjoyment of the life quality, achieving of capacities and good life [31]. There is a very complex definition for health. According to the WHO definition of health, it is a state of complete physical, mental and social well-being and not merely the absence of disease or infirmity [32]. In our study, the understanding of the health concept means the lack of physical problems of the disease and the lack of concern about the illness, in other words, after self-care, which had caused to fix all the physical problems and mental disorders. In fact, they were managing their illness in such a way that they were able to spend their daily lives normally.

It should be stated that obtaining information about it helped the adolescents to more easily accept diabetes, without self-blame and denial of illness. In this regard, Moreira and Dupas state that children considered the diabetes to be easy, and tried to gain a real understanding of its nature and therefore accepted it [22].

It is obvious that in this way, families have been considered as a source of support for adolescents so that they, as the results showed, had helped not to hide the disease and to show that it is normal, and they achieved the perceived similarities with other children. They had encouraged adolescents to participate in social activities and had no obvious change in their lifestyle, while a study shows that lifestyle will be changed in the face of chronic disease [22].

In addition, parents had tried to give some roles and responsibilities to the adolescent in families, and adolescents had undertaken independently the responsibilities for taking care of their blood sugar. Unlike the available studies, there are conflicts between the adolescent and family for his/her self-care management, and this responsibility is shared between the family and the adolescent [33]. Adolescents have been reported to develop independence and to define their identity through behaviours that form their boundaries [25]. Karlsson et al. in their study concluded that the transition towards autonomy in self-management among adolescents with type 1 diabetes was shown to be complex. Being allowed to make one’s own choices and practice decision-making aids in this transition. Realistic opportunities for a stable foundation of self-management include having the knowledge required to practice and handle different situations [3].

In this study, life satisfaction was considered as the consequence according to which adolescents with diabetes have achieved this perception that their lives are routine. Unlike our study, several studies have reported that the patients with diabetes have unfavourable lives and considered impossible to achieve a normal life [34,35]. However, in the study by Huus and Enskar, ordinary life with the disease was one of the resulting themes [26]. Also, Wennick et al. stated that diabetes is a natural element of family life in children three years after diagnosis of the disease [36]. Given the socio cultural structure and the centres providing health care services in our community, children and their families had effective benefit of existing support resources and had been able to lead their lives to normal conditions and keep it as normal [37].

The limitations of this study included, but were not exclusively, a small sample size, in terms of both the nurses who were interviewed and the facilities that were used, which thereby limited the general applicability of the results. However, these findings should be reassessed after replicating this study in other cultures and contexts.

## Conclusions

The results showed that to achieve a positive self-perception in adolescents with diabetes, the personnel providing health care services should inform them, get information about the disease and how to care it, and create sensitivity so that the clients can get the insight. In other words, “getting insight” is a primary and preparatory step to gain positive self-perception in these patients.

It is obvious that after getting insight into the practices of self-care, adolescents can independently obtain the necessary capability to provide self-care, and therefore physical, emotional, social problems, etc. can be reduced. Due to the absence or reduction of problems caused by disease, adolescents perceive similarities with healthy individuals over time. A healthy self-perception similar with normal people leads to life satisfaction in adolescents with the disease. Adolescents’ perceptions help the personnel providing health care services to facilitate the achievement of positive self-perception of health for the clients, with proper planning.
